# Morphological alternation and influence of aqueous flare in idiopathic epiretinal membrane

**DOI:** 10.1186/s40942-021-00294-9

**Published:** 2021-05-17

**Authors:** Yasuko Ikegami, Jiro Numaga, Saori Ue, Tomohiro Sano

**Affiliations:** 1 Department of Ophthalmology, Tokyo Metropolitan Geriatric Hospital, 35-2 Sakae-cho, Itabashi district, Tokyo, 173-0015 Japan; 2 Department of Ophthalmology, Graduate School of Medicine, University of Tokyo, Tokyo, Japan

**Keywords:** Epiretinal membrane, Retinal thickness, Aqueous flare, Choroidal thickness, Tangential force, Retinal folds, Optical coherence tomography

## Abstract

**Background:**

Idiopathic epiretinal membrane (iERM) is a common retinal disease in the elderly population. The exact pathogenesis of iERM is unknown. The present study aimed to evaluate the relationship between aqueous flare and morphology of iERM using swept-source optical coherence tomography (OCT).

**Methods:**

A consecutive series of 36 eyes of 33 patients with iERM and 109 control eyes of 109 patients were retrospectively examined. Aqueous flare measurements and OCT images were obtained on the same day. The average total retinal, inner retinal, outer retinal, and choroidal thicknesses were calculated using the thickness map mode with an Early Treatment Diabetic Retinopathy Study nine-zone grid that was divided into three zones. The maximum depth of the retinal folds in iERMs was manually measured. The correlation among flare value, maximum depth of folds, and retinal and choroidal thicknesses was evaluated. The morphological changes between the control eyes and the eyes with iERM in different stages were examined.

**Results:**

The result demonstrated a significant positive correlation between the aqueous flare value and total and inner retinal thicknesses in the early stage of iERM. There was a significant positive correlation between the maximum depth of folds and total and inner retinal thicknesses in the early stage of iERM, and the maximum depth of folds significantly increased in the advanced stage. The total and inner retinal thicknesses and proportion of inner retinal thickness significantly increased as the stage of iERM progressed.

**Conclusions:**

The aqueous flare value was associated with retinal thickness in the early stage of iERM, which supports the idea that inflammation or breakdown of blood–ocular barrier is involved in the process of iERM formation. The maximum retinal folds increased as the stage of iERM progressed and retinal thickness increased, which indicates that the tangential force increases as the iERM stage progresses.

## Background

Epiretinal membrane (ERM) is a fibrocellular proliferation at the vitreoretinal interface on the inner retinal surface and leads to visual impairment or metamorphopsia. It is a common macular disease in elderly individuals and affects millions of people worldwide [1–3]. ERMs are classified as “idiopathic” if they are not associated with any other ocular abnormality. The exact pathogenesis of idiopathic ERM (iERM) remains unclear. However, there is a growing need for techniques to evaluate ERM severity and predict outcomes.

It is generally believed that posterior vitreous detachment (PVD) plays a critical role in the pathogenesis of iERM [3–5]. The vitreous cortical remnants on the retinal surface after PVD, which contains hyalocytes, provides a medium for the proliferation and transdifferentiation of glial cells. These hyalocytes stimulate Müller cells to send the process through an intact inner limiting membrane (ILM) to form scaffolding. Activated Müller glial cells contribute to ERM formation [6]. PVD and chronic irritation of glial cells can cause a local release of factors that induce cell gliosis and inflammatory process [7, 8]. ERM formation requires cell migration and proliferation, extracellular matrix formation, and tissue contraction. Recently, researchers have elucidated that growth factors and cytokines are involved in iERM formation and inflammatory mechanisms are implicated in iERM formation [5, 9–13].

The aqueous flare value measured by a laser flare cell meter reflects the degree of inflammation and functions of the blood–aqueous barrier and blood–retinal barrier [14, 15], and it has been reported that the aqueous flare value correlates with the cytokine levels in aqueous humor [16]. A laser flare cell meter allows the detection of subclinical alterations in the aqueous humor. Some groups reported that the increased flare value in posterior uveitis may not always be related to the breakdown of the blood–aqueous barrier but may result from the entry of proteins into the aqueous humor from the posterior segment. Proteins may enter the aqueous humor from the posterior segment of the eyes, and the flare value may reflect not only inflammation of the anterior segment but also that of the posterior segment of the eye [17].

Recently, with the development of high-resolution optical coherence tomography (OCT), microstructural analysis has become possible and allows better visualization and measurement of retinal layers. ERM formation induces intraretinal morphological change. Traction by ERM influences the morphology and thickness of each retinal layer beneath the membrane. Previously, several studies have examined the retinal and choroidal morphological features of iERM [18, 19]. However, the relationship between the morphology and pathogenesis of iERM is still controversial. The present study aimed to evaluate the aqueous flare and retinal thickness (RT) of the eyes in patients with iERM and examine the relationship between ocular inflammation and thickness of each retinal layer and morphology of the iERM.

## Methods

### Patients and study design

We retrospectively reviewed the records of consecutive patients who were scheduled for cataract surgery and underwent OCT and aqueous flare measurements between August 2018 and December 2018, and recruited patients with iERM and controls without ERM. Thirty-six eyes of 33 patients (26 women and 7 men) with iERM and 109 control eyes of 109 patients (73 women and 36 men) without iERM were selected in this study. The mean ages were 76.17 ± 6.16 years (range 67–89 years) and 77.81 ± 7.01 years (range, 61–95 years) for the patient and control groups, respectively. ERM was diagnosed based on the OCT findings and defined as a highly reflective membrane on the retinal surface that proliferated on the surface of the ILM in 12 radial lines of OCT images. The inclusion criterion was adequate OCT linear scans. The exclusion criteria were as follows: axial length > 26.5 or < 21 mm in the affected eye; significant media opacities, especially cataract that interferes with the aqueous flare measurement; diagnosis or history of any ocular diseases that might influence the study results, such as glaucoma, uveitis, age-related macular degeneration, retinal vascular disease, inflammatory eye disease, neurodegenerative disease, and macular or lamellar holes in the eyes; history of retinal surgery, photocoagulation or trauma; diabetes.

All patients underwent comprehensible ophthalmic examinations, including slit-lamp microscopy examination, intraocular pressure measurement, fundoscopy, and axial length measurement. Axial length was measured using the IOL Master® V3.01 (Carl Zeiss Meditec, Jena, Germany). The institutional ethics committee of Tokyo Metropolitan Geriatric Hospital approved the present study. All research and measurements adhered to the tenets of the Declaration of Helsinki.

### Swept-source OCT: image acquisition

**Fig. 1:**
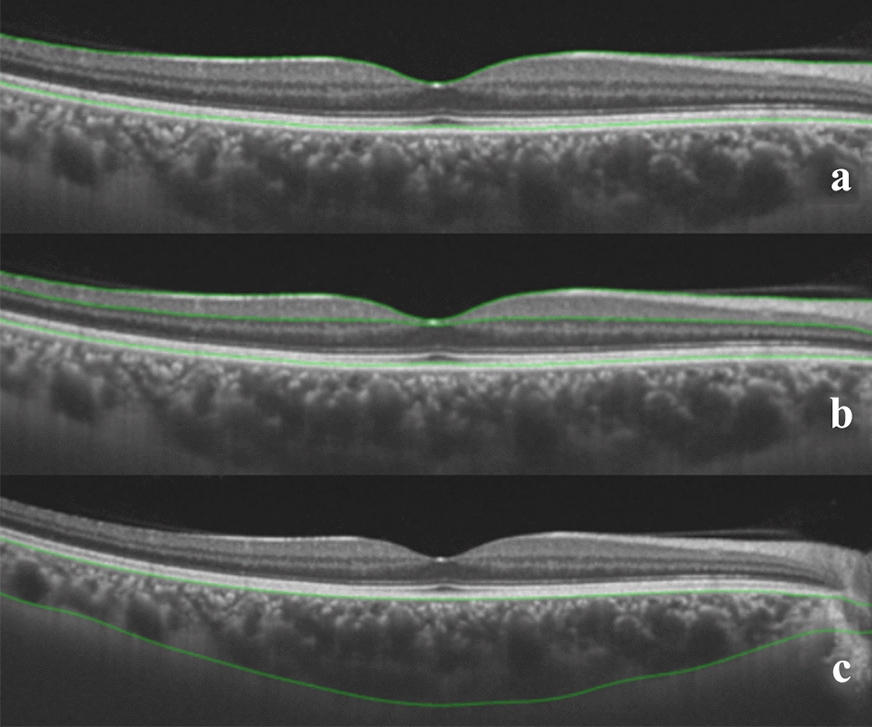
Automatic Retinal segmentation depicted by OCT. **a** (total retina, ILM-RPE). **b** (inner retina, ILM-INL; outer-retina, INL-RPE). **c** (choroid, BM-choroidal-scleral boundary). *ILM* internal limiting membrane, *RPE* retinal pigment epithelium, *INL* inner nuclear layer, *BM* basement membrane

During the same visit, all study subjects underwent swept-source (SS)-OCT (DRI Triton, Topcon, Tokyo, Japan), which contains a 1050 nm wavelength swept light source and has a scanning speed of 100,000 A-scans per second. We obtained a 12 radial pattern scan centered on the fovea from each eye. The OCT device automatically segments the layers using a built-in segmentation algorithm. The total retina, inner retina, and outer retina and choroid were defined as the area from the ILM to the inner surface of the retinal pigment epithelium (RPE), area from the ILM to the inner surface of the inner nuclear layer (INL), area from the INL to the inner surface of the RPE, and area from the outer border of the basement membrane to the choroid-scleral boundary, respectively (Fig. 1). The average total RT, inner RT, outer RT, and choroidal thickness (CT) were calculated automatically. If the lines were misaligned, they were adjusted manually.

The ERM appears as a thin smooth hyperreflective band between the retina and vitreous humor. This band lies on the retinal surface and is sometimes partially separated from the inner retinal surface. In areas with a hyporeflective gap between the ERM and inner border of the retina, we set a segmentation line of the inner border of the retina on the outer margin of hyporeflective space. In areas without hyporeflective spaces, we used the outer margin of the ERM as the inner boundary of the retina. This method is described by Koo et al. [18].

**Fig. 2:**
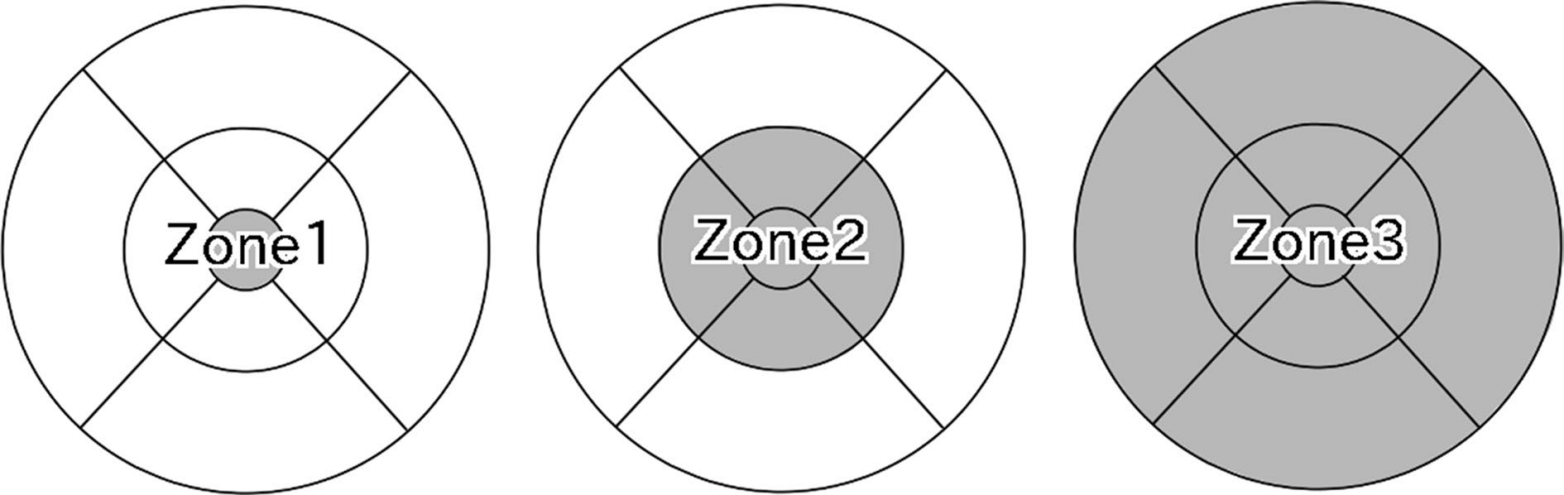
Three zones defined using the ETDRS grid. The ETDRS grid was divided into three zones: a central circle with a diameter of 1 mm (zone 1), a parafoveal circle with a diameter of 3 mm (zone 2), and an outer circle of diameter of 6 mm (zone 3)

An Early Treatment Diabetic Retinopathy Study (ETDRS) map (6 × 6 mm) was constructed automatically for the RT and CT using the volume scan on SS-OCT. The average RT was evaluated based on three zones defined using the ETDRS grid: a central circle with a diameter of 1 mm (zone 1), a parafoveal circle with a diameter of 3 mm (zone 2), and an outer circle of diameter of 6 mm (zone 3) (Fig. 2). All OCT measurements were conducted between 10:00 AM and 1:00 PM to reduce the effects of diurnal variation on their measurements.

### Grading of ERM and measurement of the maximum depth of retinal folds

**Fig. 3:**
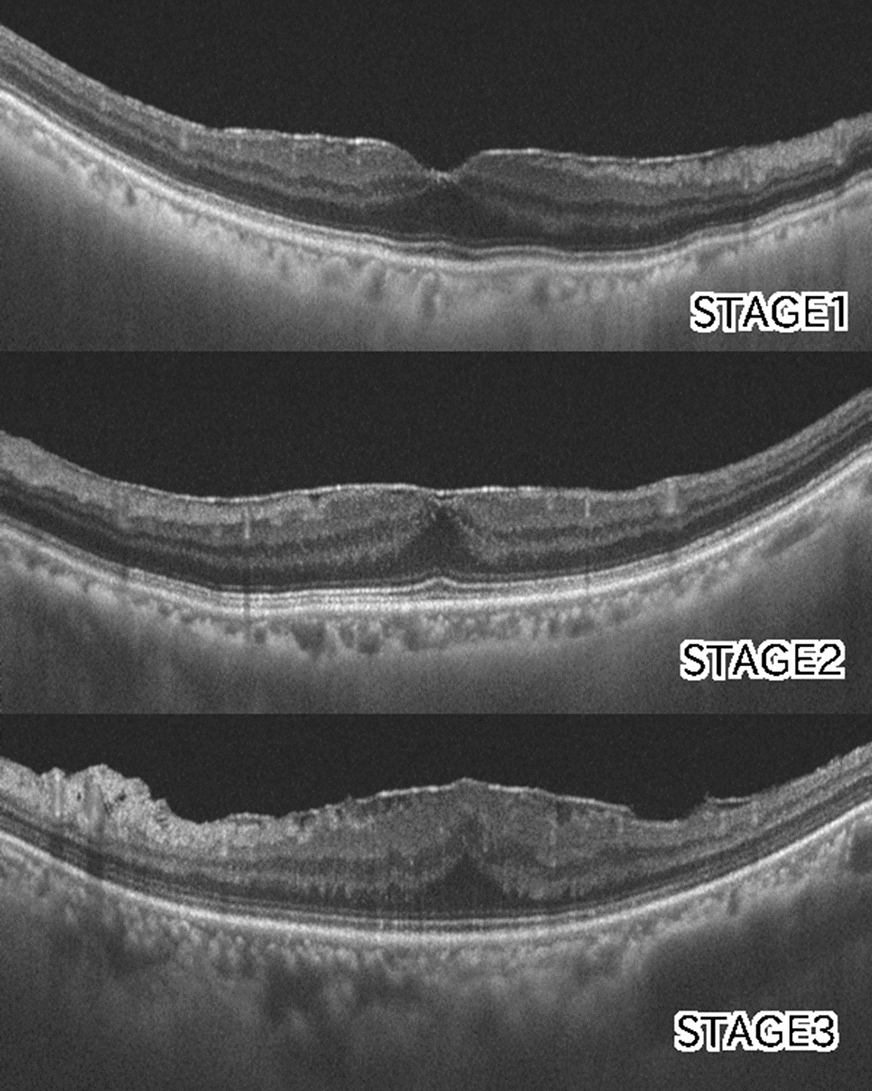
Optcal coherentce tomography staging of idiopathic epiretinal membrane by Covetto. Stage 1, presence of the foveal pit and well-defined retinal layers; Stage 2, absence of the foveal pit and presence of well-defined retinal layers; Stage 3, absence of the foveal pit and well-defined retinal layers and presence of ectopic inner foveal layers

**Fig. 4:**
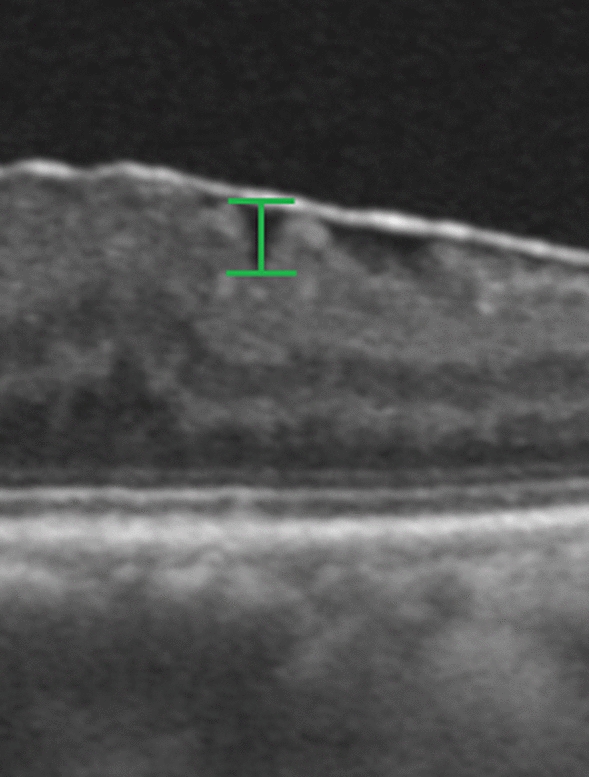
Measurement of maximum length of the retinal folds. The depths of the folds were measured from the top of the subsequent folds to the bottom of the subsequent folds, and among these, the maximum depth was determined

The extent of ERM was graded based on the system proposed by Govetto [20]: stage 1, presence of the foveal pit and well-defined retinal layers; stage 2, absence of the foveal pit and presence of well-defined retinal layers; stage 3, absence of the foveal pit and well-defined retinal layers and presence of ectopic inner foveal layer; and stage 4, absence of the foveal pit and disrupted retinal layers and presence of ectopic inner foveal layer (Fig. 3). To quantitatively analyze the strength of retinal traction, we measured the maximum depth of folds of the inner margin of the retinal nerve fiber layer within a 6 mm-diameter circle centered at the fovea, in 12 lines of radial pattern scans. The depths of the folds were measured from the top of the subsequent folds to the bottom of the subsequent folds, and among these, the maximum depth was determined. We magnified the image to the maximum and measured the folds perpendicularly to acquire an accurate measurement (Fig. 4).

### Aqueous flare measurement

**Fig. 5:**
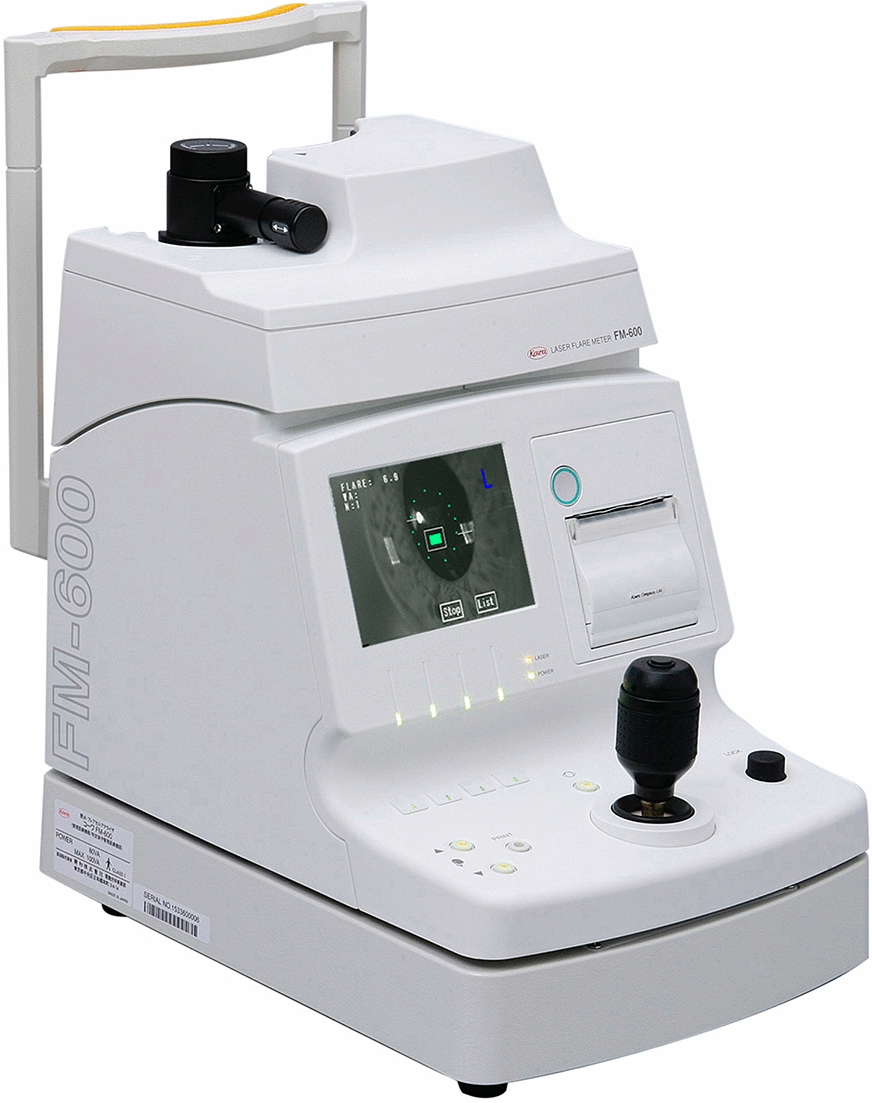
Laser flare cell meter (FM-600 Kowa Company, Nagoya, Japan)

The aqueous flare value was determined on the same day of OCT image acquisition using a laser flare cell meter (FM-600 Kowa Company, Nagoya, Japan) (Fig. 5). Five consecutive flare readings with background scatter of < 15% were acquired and averaged for each eye. The results are expressed as photon counts per millisecond. The flare intensity was measured 30–60 min after the application of 0.5% tropicamide and 0.5% phenylephrine hydrochloride eye drops.

### Statistical analysis

The correlation between aqueous flare and RT and CT of the eyes in each stage was determined by Pearson’s correlation analysis. The correlation between the maximum depth of the retinal folds and RT and CT of the eyes in each stage was determined via Pearson’s correlation analysis. To compare the means of RT and CT and flare between three groups, we used a one-factor analysis of variance or the Kruskal–Wallis test, as appropriate. Post hoc comparisons were tested via the Tukey or Steel–Dwass correction. The data were expressed as mean ± standard deviation. Normally distributed data were analyzed using the Kolmogorov–Smirnov test. A *P* value of < 0.05 was considered statistically significant. Statistical analyses were conducted using EZR (Saitama Medical Center, Jichi Medical University, Saitama, Japan), which is a graphical user interface for R (The R Foundation for Statistical Computing, Vienna, Austria) [21].

## Results

This retrospective study included 36 eyes with iERM and 109 control eyes without iERM. There was no significant difference in age (p = 0.213) and sex (p = 0.296) between patients with iERM and controls; however, there was a significant difference in axial length (p = 0.0416). Of 36 eyes with iERM, 26 eyes were in stage 1, 8 eyes were in stage 2, and 2 eyes were stage 3. In analyzing the result, the 10 values in stages 2 and 3 were treated as the same group. The mean ages of the patients with iERM of stage 1 and iERM of stages 2 and 3 were 76.38 ± 6.22 and 75.60 ± 6.31 years, respectively. The mean axial lengths were 24.17 ± 1.22 and 24.26 ± 1.34 mm, respectively. There was no significant difference in age (p = 0.949), sex (p = 0.259), and axial length (p = 0.980) between eyes in stage 1 and stages 2 and 3 (Table 1).

**Table 1:** Characteristics of groups and comparison of mean thickness of each layer in eyes with and without iERM

	eyes with iERM	control eyes without ERM	*P* value*	iERM stage 1	*P* value**	iERM stage 2,3	*P* value***
	n = 36	n = 109		n = 26		n = 10	
Age (years)	76.17 ± 6.16	77.81 ± 7.01	0.213^a^	76.38 ± 6.22	0.609^b^	75.60 ± 6.31	0.949^b^
Gender (male/female)	8/28	36/73	0.296^d^	4/22	0.182^d^	4/6	0.259^d^
Axial length (mm)	24.19 ± 1.23	23.73 ± 1.14	** < 0.05**^a^	24.17 ± 1.22	0.204^b^	24.26 ± 1.34	0.980^b^
Aqueous flare (pc/ms)	8.09 ± 3.97	7.17 ± 2.94	0.207^a^	8.06 ± 4.37	0.841^b^	8.16 ± 2.87	0.839^b^
Maximum length of retinal folds (µm)				26.92 ± 19.05		50.60 ± 26.58	** < 0.01**^a^
Total retinal thickness (µm) Mean ± SD							
Zone 1	291.75 ± 49.48	232.63 ± 23.75	** < 0.0001**^a^	269.65 ± 35.91	** < 0.0001**^c^	349.20 ± 29.39	** < 0.001**^c^
Zone 2	315.55 ± 23.22	287.03 ± 17.28	** < 0.0001**^a^	307.40 ± 17.97	** < 0.0001**^b^	336.76 ± 22.54	** < 0.0001**^b^
Zone 3	281.87 ± 16.09	262.99 ± 14.48	** < 0.0001**^a^	278.46 ± 14.27	** < 0.0001**^b^	290.76 ± 17.83	0.0661^b^
Inner retinal thickness (µm) Mean ± SD							
Zone 1	71.53 ± 22.19	49.44 ± 12.51	** < 0.0001**^a^	62.58 ± 15.54	** < 0.001**^c^	94.80 ± 20.27	** < 0.001**^c^
Zone 2	119.47 ± 18.18	100.17 ± 11.46	** < 0.0001**^a^	114.63 ± 15.96	** < 0.0001**^c^	136.73 ± 18.27	** < 0.05**^c^
Zone 3	112.30 ± 12.68	99.75 ± 11.12	** < 0.0001**^a^	110.10 ± 11.61	** < 0.001**^b^	117.99 ± 14.19	0.156^b^
Outer retinal thickness (µm) Mean ± SD							
Zone 1	220.22 ± 31.35	183.19 ± 16.88	** < 0.0001**^a^	207.08 ± 25.82	** < 0.0001**^c^	254.40 ± 13.22	** < 0.001**^c^
Zone 2	196.08 ± 15.81	186.87 ± 11.45	** < 0.01**^a^	192.77 ± 16.94	** < 0.05**^c^	204.69 ± 7.83	** < 0.05**^c^
Zone 3	169.58 ± 8.93	163.24 ± 10.24	** < 0.01**^a^	168.35 ± 9.15	0.0512^b^	172.77 ± 7.87	0.458^b^
% Inner retinal thickness (%) Mean ± SD							
Zone 1	24.12 ± 4.14	21.08 ± 4.08	** < 0.001**^a^	23.05 ± 3.75	0.0656^b^	26.90 ± 3.98	** < 0.05**^b^
Zone 2	37.77 ± 4.21	34.84 ± 2.84	** < 0.001**^a^	37.26 ± 4.54	** < 0.001**^c^	39.07 ± 3.02	0.169^c^
Zone 3	39.76 ± 2.79	37.76 ± 2.79	** < 0.01**^a^	39.49 ± 2.81	** < 0.05**^b^	40.48 ± 2.76	0.653^b^
% Outer retinal thickness (%) Mean ± SD							
Zone 1	75.88 ± 4.14	78.92 ± 4.08	** < 0.001**^a^	76.94 ± 3.75	0.0656	73.10 ± 3.98	** < 0.05**^b^
Zone 2	62.23 ± 4.21	65.16 ± 2.84	** < 0.001**^a^	62.74 ± 4.54	** < 0.001**^c^	60.93 ± 3.02	0.169^c^
Zone 3	60.23 ± 2.79	62.12 ± 3.10	** < 0.01**^a^	60.51 ± 2.81	** < 0.05**^b^	59.52 ± 2.76	0.653^b^
Choroidal thickness (µm) Mean ± SD							
Zone 1	210.39 ± 78.52	212.74 ± 57.58	0.869^a^	202.88 ± 58.79	0.611^c^	229.90 ± 117.43	0.983^c^
Zone 2	204.98 ± 73.19	208.25 ± 57.04	0.782^a^	198.59 ± 56.29	0.613^c^	221.61 ± 107.74	0.976^c^
Zone 3	193.86 ± 64.59	197.68 ± 52.49	0.722^a^	187.63 ± 50.51	0.627^c^	210.05 ± 92.90	0.999^c^

Bold *P* values denote statistical signigficance. iERM, idiopatic epiretinal membrane

^*^Between eyes with iERM and control eyes

^**^Between control eyes and eye with iERM in stage1

^***^Between eyes with iERM stage 1 and eyes with iERM in stage 2 and 3

^a^Unpaired t-test

^b^Unpaired t-test with Tukey post-test

^c^Mann-Whitney test with Steel–Dwass post-test

^d^Fisher-test

Aqueous flare value of the eyes with iERM exhibited a significant positive correlation with the total RT, inner RT, and % inner RT (percentage of inner RT of the total RT); however, the flare value exhibited a significant negative correlation with % outer RT. The flare value had no significant correlation with outer RT, CT, and the maximum depth of the retinal folds. In control eyes without iERM and eyes with iERM in stage 2 or 3, there was low significant correlation between aqueous flare value and RT or CT (Table 2).

**Table 2:** Correlation between aqueous flare and retinal and choroidal thickness in eyes with or without ERM

	Control without ERM		iERM stage 1		iERM stage 2,3	
	n = 109		n = 26		n = 10	
	r*	*P* value	r*	*P* value	r*	*P* value
Total retinal thickness						
Zone 1	0.167	0.083	0.544	** < 0.01**	0.389	0.266
Zone 2	0.152	0.0682	0.350	0.0793	− 0.0663	0.856
Zone 3	0.184	0.271	0.511	** < 0.01**	− 0.0209	0.954
Inner retinal thickness						
Zone 1	0.287	** < 0.01**	0.667	** < 0.001**	0.405	0.246
Zone 2	0.241	** < 0.01**	0.655	** < 0.001**	0.054	0.882
Zone 3	0.225	** < 0.01**	0.633	** < 0.001**	0.0507	0.889
Outer retinal thickness						
Zone 1	0.225	** < 0.01**	0.356	0.0744	0.244	0.496
Zone 2	− 0.0287	0.732	− 0.0246	0.226	− 0.186	0.606
Zone 3	0.0243	0.771	− 0.0432	0.835	− 0.242	0.501
%Inner retinal thickness						
Zone 1	0.210	** < 0.05**	0.503	** < 0.01**	0.409	0.241
Zone 2	0.235	** < 0.01**	0.574	** < 0.01**	0.117	0.748
Zone 3	0.175	** < 0.05**	0.564	** < 0.01**	0.138	0.703
%Outer retinal thickness						
Zone 1	− 0.21	** < 0.05**	− 0.503	** < 0.01**	− 0.409	0.241
Zone 2	− 0.235	** < 0.01**	− 0.564	** < 0.01**	− 0.117	0.748
Zone 3	− 0.174	** < 0.05**	− 0.574	** < 0.01**	− 0.138	0.703
Choroidal thickness						
Zone 1	− 0.194	** < 0.05**	− 0.265	0.191	0.194	0.592
Zone 2	− 0.190	** < 0.05**	− 0.253	0.212	0.238	0.507
Zone 3	− 0.223	** < 0.01**	− 0.316	0.115	0.228	0.527

Bold *P* values denote statistical significance

^*^Pearson correlation coefficient

There was a significant positive correlation between the maximum depth of the retinal folds and total and inner RT; however, there was no significant correlation between the maximum depth of the retinal folds and flare value, outer RT, and CT. When the stage of iERM advanced to stage 2 or 3, the correlations between the maximum length of the retinal folds and RT or CT were not significant (Table 3).

**Table 3:** Correlation with maximum length of retinal fold and retinal and choroidal thickness and aqueous flare in eyes with iERM

	iERM stage 1		iERM stage 2,3	
	n = 26		n = 10	
	r*	*P*	r*	*P*
Total retinal thickness				
Zone 1	0.232	0.254	0.265	0.459
Zone 2	0.472	** < 0.05**	− 0.0793	0.828
Zone 3	0.626	** < 0.001**	− 0.403	0.249
Inner retinal thickness				
Zone 1	0.110	0.591	− 0.0679	0.852
Zone 2	0.446	** < 0.05**	− 0.0721	0.843
Zone 3	0.484	** < 0.05**	− 0.316	0.374
Outer retinal thickness				
Zone 1	0.273	0.177	0.485	0.155
Zone 2	0.0813	0.693	− 0.060	0.869
Zone 3	0.362	0.069	− 0.345	0.328
Choroidal thickness				
Zone 1	− 0.146	0.477	− 0.134	0.712
Zone 2	− 0.149	0.469	− 0.111	0.760
Zone 3	− 0.179	0.383	− 0.110	0.761
Aqueous flare	0.350	0.0798	0.211	0.558

Bold *P* values denote statistical significance

^*^Pearson correlation coefficient

We compared the eyes with iERM and control eyes without ERM. The total RT, inner RT, outer RT, and % inner RT of the eyes with iERM were significantly higher than those of the control eyes. However, % outer RTs of the eyes with iERM were significantly lower than those of the control eyes (Table 1). During the comparison between stage 1 and stages 2 and 3, it was observed that the total RT, inner RT and outer RT of the eyes with iERM of stage 2 and 3 were significantly higher than in eyes with iERM of stage 1, except for zone 3. However, there was a significant difference in % inner RT only in zone 1. The maximum length of the retinal folds significantly increased from stage 1 to stages 2 and 3. There was no significant difference in CT and aqueous flare value between eyes with iERM and control eyes and between eyes with iERM stage 1 and eyes with iERM stages 2 and 3 (Table 1).

## Discussion

The present study found that the aqueous flare value was significantly correlated with the total RT (zones 1 and 3) and inner RT in eyes with iERM in stage 1. The correlation coefficient was not significant in stages 2 and 3. The correlation coefficient was low in the control eyes. The fact that an increase in the aqueous flare associated with the increase in the RT implied the relationship between iERM progression and inflammation or breakdown of the blood–ocular barrier. In fact, several researchers supported the role of inflammation in the pathogenesis of iERM.

One of the facts that indicate the involvement of inflammation in iERM pathogenesis is the presence of inflammatory cells in iERMs. The presence of proliferation of inflammatory cells, such as macrophages, lymphocytes, neutrophils, and monocytes in iERMs, has been previously noted [9, 12, 22]. Other previous studies have reported that specific cytokines were expressed in iERMs, such as vascular endothelial growth factor, interleukin-6, transforming growth factor-beta, and connective tissue growth factor [10, 11, 23].

Furthermore, in several studies, the result of the actual measurement of protein levels in vitreous and aqueous samples from human eyes with iERMs showed elevated levels of various proteins, and they concluded that iERMs were characterized by a complicated process that involved changes in the levels of numerous proteins [13, 24]. Other studies showed a significantly higher level of cytokines in vitreous samples from patients with iERM [8, 10, 11, 23, 25]. These activated cytokine profiles, which existed in the vitreous fluid of patients with ERM, are proinflammatory and play a major role in immunomodulation and inflammation [26]. Other studies reported that in the iERM vitreous samples, complement components, inflammation-related proteins, and matrix metalloproteinase exist at higher levels [10].

On the other hand, several researchers suggested that cytokines and growth factors that are protein components in the vitreous humor are crucial in the progression of iERM. That is, in the process of iERM formation, several cytokines and growth factors might promote increased fibroblast migration and deposition, differentiation into myofibroblasts, and contraction of the extracellular matrix [3, 8, 10, 25]. The contraction of myofibroblasts within the ERM has been proposed to exert a tangential traction, which could result in retinal thickening and folding [3, 27].

Furthermore, in the relationship between ERM formation and inflammation, Müller cells play an important role. The role of Müller cells in ERM formation, especially in the early stage, involves the interaction of inflammatory and glial cells. After partial PVD, vitreous fibers adhering to Müller cells at sites of vitreoretinal attachment exert tractional forces onto the cells; this causes mechanical stress to Müller cells [5, 6] and activates the cells in response to vitreal growth factors and cytokines and results in cellular hypertrophy, proliferation, and vascular leakage, which induce contraction of Müller cells and lead to retinal fold formation [6, 28, 29]. Commonly, retinal disease is associated with reactive gliosis of three kinds of retinal glial cells, namely, Müller cells, astrocytes, and microglia. Activated microglial cells show morphological alterations and proliferate and migrate throughout the whole retinal tissue and initiate inflammatory processes and tissue repair [6]. Based on the abovementioned reasons, we speculated that during ERM formation, the activities of glial cells are related to the increased inflammation and increase in RT.

However, we also speculated other contradictory possible reasons for the increase in aqueous flare in proportion to the increase in RT; we suspected that the inflammation factors that originally existed in the eyes might be the cause of the iERM. Namely, the eyes with originally increased aqueous flare might tend to easily exhibit ERMs and increased RT. In fact, it has been suggested for quite some time that inflammation induced ERM formation. In the present study, we carefully evaluated the enrolled eyes and found no inflammation-related disease in these eyes; however, some eyes might have subclinical inflammation or history of inflammatory disease, which influenced ERM formation. Mcleod et al. suggested in their studies that ERM formation was typically a response to a chronic stimulus, often inflammatory event, and suggested that idiopathic age-related ERM formation was unlikely to be related simply to PVD [30].

In the present study, the total RT, inner RT, and % inner RT were significantly greater in eyes with iERM than in control eyes without ERM. However, the proportion of outer RT was lower in eyes with iERM than in control eyes. These results were consistent with those of previous studies. Several studies reported that all retinal layers, including the inner retina (sum of RNFL, GCL, and IPL thicknesses), were thicker in eyes with ERM than in normal eyes and speculated that this might have resulted from retinal contraction and edema rather than actual tissue gain [18, 31]. Furthermore, Koo et al. demonstrated that, in the eye with ERM, the proportion of thickness of the inner retina and INL were significantly higher than those of the fellow eye. Although all layers of the ERM retina were thicker than those of the normal retina, the ONL, which was located on the farther portion from the vitreous, was relatively thin [18]. They also described that, although the thickness increases by traction of ERM, the tangential force originates from the surface of the retina (near the inner retina) and not in the outer retina or the choroid [18]. Lee et al. reported that greater RT might be associated with greater traction hence more severe retinal ganglion cell (RGC) damage [32].

In the present study, we also manually measured the maximum depth of the deepest retinal folds for a quantitative assessment of the strength of retinal traction. Hirano et al. revealed that when folds are generated in thin elastic membranes, the maximum amplitude of the fold becomes larger as the compression stress on the membrane increases, and they described the maximum depth of the retinal folds as an indicator to estimate the strength of retinal traction force caused by ERM [33]. We found that the maximum depth of the retinal folds correlated with total RT and inner RT of zones 2 and 3 in stage 1, which were consistent with the result of Hirano et al.’s study [33]. Additionally, in the present study, the maximum depth of the retinal folds increased significantly from stage 1 to stages 2 and 3.

The vitreoretinal traction forces can be divided into two types: anterior–posterior force and tangential force [3]. Anteroposterior vitreoretinal traction is caused by persistent vitreoretinal adhesions caused by an incomplete PVD, whereas tangential traction is caused by contractive ERMs as a result of progressive fibrocellular proliferation at the vitreal side of the ILM [22]. ERM tangential traction affects the inner retina, causing retinal wrinkling, subsequent RGC damage, and impairment of inner retinal structural integrity [32]. The inner retinal changes might result from the combination of both physical displacement of the inner retinal layers and Müller cell-driven proliferation [7]. The traction on the retinal surface may induce Müller cell gliosis [5], and Müller cells play an interaction of inflammatory and glial cells. This reactive gliosis with vertical glial proliferation from ERM throughout the retina causes greater tangential traction along with intraretinal changes involving deeper retinal layers [34, 35]. Once ERMs are formed, the contraction of the membranes may produce further retinal folds [35]. In the present study, the maximum depth of the retinal folds, which indicates the tangential force, was associated with RT and increased as the ERM stage progressed. This result can be explained by the extent of traction force during the process of iERM formation as described above.

Several researchers reported that chronic or stronger traction forces might affect the outer retinal layer, including the photoreceptor layer. Gomes et al. suggested that chronic untreated ERMs could induce permanent changes in the outer layers of the retina secondary to longstanding traction forces [36]. Recently published studies support the correlation between visual acuity and state of the IS/OS junction of photoreceptors and metamorphopsia and thickness of the INL [31, 37]. Thus, advancement in the disease results in significantly reduced visual function. ERM is only symptomatic if the outer retinal layer, including the photoreceptor layer, is involved. The initial formation of this epiretinal tissue does not usually cause any clinically important reduction in vision. Therefore, the prevention or treatment before the onset of the symptom is crucial. The increase in RT in eyes with ERM might be another threatening factor of visual impairment and indicator of disease progression.

The present study had several limitations. First, this was a retrospective study on a limited number of participants. There is a possibility of patient selection bias because of the retrospective nature of the study. The present study mainly included elderly patients because this was an institutional study in a geriatric hospital that was originally built for elderly patients. The stage of iERM included in the present study was limited to earlier stages 1 to 3. We measured only the inner and outer retina and not each layer of the retina, and the segmentation software used in the present study included the INL in the outer retina. We could not identify which layers were responsible for the increase in the inner layer. Lastly, when measuring the depth of the fold, we had to manually define these parts. We evaluated only the vertical depth and did not include the horizontal spread boundary.

## Conclusions

The aqueous flare value was positively correlated with RT in the early stage of iERM, which suggests that inflammation or breakdown of blood–ocular barrier is involved in the process of iERM formation. The maximum length of the retinal folds increased as the stage of iERM advanced and as the RT increased, which indicates that the tangential force increases as iERM progresses. Taken together, these findings suggest that inflammation affects the increase in volume, formation of retinal folds, and tangential force in iERM.

## Data Availability

The datasets used and analyzed during the present study are available from the corresponding author on reasonable request.

## Abbreviations

OCT: Optical coherence tomography
iERM: Idiopathic epiretinal membrane
ETDRS: Early Diabetic Retinopathy Study
ERM: Epiretinal membrane
PVD: Posterior vitreous detachment
RT: Retinal thickness
ILM: Internal limiting membrane
RPE: Retinal pigment epithelium
INL: Inner nuclear layer
BML: Basement membrane
IPL: Inner plexus layer
CT: Choroidal thickness
IS/OS: Inner segment/outer segment
